# A 5‐gene prognostic nomogram predicting survival probability of glioblastoma patients

**DOI:** 10.1002/brb3.1258

**Published:** 2019-03-11

**Authors:** Lingchen Wang, Zhengwei Yan, Xiaona He, Cheng Zhang, Huiqiang Yu, Quqin Lu

**Affiliations:** ^1^ Department of Biostatistics and Epidemiology, School of Public Health Nanchang University Nanchang China; ^2^ Jiangxi Provincial Key Laboratory of Preventive Medicine Nanchang University Nanchang P.R. China; ^3^ Center for Experimental Medicine The First Affiliated Hospital of Nanchang University Jiangxi China

**Keywords:** differentially expressed genes, glioblastoma, overall survival, prediction method, prognostic nomogram, SCR_001175, SCR_001905, SCR_003193, SCR_005012, SCR_006472, SCR_006786, SCR_010943, SCR_012802, survival probability

## Abstract

**Background:**

Glioblastoma (GBM) remains the most biologically aggressive subtype of gliomas with an average survival of 10 to 12 months. Considering that the overall survival (OS) of each GBM patient is a key factor in the treatment of individuals, it is meaningful to predict the survival probability for GBM patients newly diagnosed in clinical practice.

**Material and Methods:**

Using the TCGA dataset and two independent GEO datasets, we identified genes that are associated with the OS and differentially expressed between GBM tissues and the adjacent normal tissues. A robust likelihood‐based survival modeling approach was applied to select the best genes for modeling. After the prognostic nomogram was generated, an independent dataset on different platform was used to evaluate its effectiveness.

**Results:**

We identified 168 differentially expressed genes associated with the OS. Five of these genes were selected to generate a gene prognostic nomogram. The external validation demonstrated that 5‐gene prognostic nomogram has the capability of predicting the OS of GBM patients.

**Conclusion:**

We developed a novel and convenient prognostic tool based on five genes that exhibited clinical value in predicting the survival probability for newly diagnosed GBM patients, and all of these five genes could represent potential target genes for the treatment of GBM. The development of this model will provide a good reference for cancer researchers.

## 1 INTRODUCTION

Malignant gliomas are the most frequent and lethal brain tumors worldwide (Louis et al., 2007), and they account for approximately 80% of primary malignant brain tumors (Omuro & Deangelis, 2013). These tumors grow rapidly, recur easily (Meir et al., 2010) and represent a leading cause of cancer‐related deaths in adults and children (Natesh et al., 2015). Gliomas are categorized as low grade glioma (LGG) and high grade glioma (HGG) (Wang et al., 2009; Wang & Jiang, 2013). HGG includes grade IV glioblastoma (GBM) (Brennan et al., 2013), the most biologically aggressive subtype of glioma with an average survival of 10 to 12 months (Ning, Hao, Feng, & Zou, 2017). Invasion and neo‐angiogenesis are the hallmarks of GBM (Cooper et al., 2012). The current standard of care for GBM patients is surgical resection followed by adjuvant radiation therapy and chemotherapy with the oral alkylating agent temozolomide (Parsons et al., 2008), which minimally contributes to the prognosis of GBM by only prolonging the median survival to 15 months (Stupp et al., 2005). Unfortunately, life‐threatening tumor recurrence is inevitable in the vast majority of patients given the best available treatments (Wang et al., 2017). Thus, it is necessary to develop novel treatments to improve the prognosis of GBM. Gene targeting provides new hope for GBM patients. And in recent decades, considerable effort has been placed on the identification of genetic alterations in GBMs that might help to define GBM patients with varied prognoses or responses to specific therapies (Mellinghoff et al., 2005). However, very few tumor‐specific targets have been identified, tested, and validated for clinical development (Ge et al., 2017). Hence, it is urgent to develop methods to identify reliable therapeutic gene targets that could enable earlier prognostic evaluation and better therapeutic strategies.

The overall survival (OS) of each GBM patient is a critical factor to devise a personal treatment plan. Therefore, it is important to develop a reliable tool to predict the survival probability for newly diagnosed GBM patients in clinical practice. Given the remarkable development of high‐throughput technologies for the profiling of genome‐wide methylation and expression, such as methylation microarray and MeDip‐seq, and RNA‐seq, and the publicly available datasets around the world (He, Zhang, Shi, & Lu, 2018; Ning et al., 2017), we were able to analyze world‐wide data. In previous cancer studies, a risk score system based on genes was applied to identify cancer patients with a high risk of mortality (Chen et al., 2017). Here, we developed a new prognostic tool to make the prediction method more convenient and intuitive.

We aimed to generate an easy and effective prognostic tool based on several genes and other factors that may affect OS. Using the TCGA dataset and two independent GEO datasets, we identified 168 genes that were associated with OS and differentially expressed between GBM tissues and adjacent normal tissues. Furthermore, five of these genes were selected by a robust likelihood‐based survival modeling approach to generate a gene prognostic nomogram. We used an independent dataset to validate the effectiveness of the nomogram, demonstrating its clinical value for predicting the survival probability for newly diagnosed GBM patients.

## 2 MATERIAL AND METHODS

### 2.1 GBM dataset from TCGA and survival analyses

We first downloaded the gene expression dataset and the clinical information of GBM patients including 168 samples from TCGA (The Cancer Genome Atlas, RRID:SCR_003193) cohort using the Illumina RNA Sequencing method. The normalization was performed using the “calcNormFactors” function from the R package “edgeR” (edgeR, RRID:SCR_012802). This function normalizes for RNA composition by finding a set of scaling factors for the library sizes that minimize the log‐fold changes between the samples for most genes using a trimmed mean of Mvalues (TMM) (Robinson & Oshlack, 2010). After normalization, the gene expression data of each sample were matched with the OS information. Statistical analyses were performed using R (R Project for Statistical Computing, RRID:SCR_001905). Kaplan‐Meier curves for high and low expression groups of each gene were plotted using the “survfit” function from the R package “survival”. Calculated using the “survdiff” function, a *p*‐value of log rank test less than 0.05 was considered statistically significant.

### 2.2 Analysis of differentially expressed genes

We identified the differentially expressed genes (DEGs) within the TCGA cohort, using the “exactTest” function from the R package “edgeR” which is based on the quantile‐adjusted conditional maximum likelihood (qCML) method (Robinson & Smyth, 2008), via the thresholds of fold change greater than 1 or less than −1 and adjusted *p* < 0.05. This dataset included 168 samples from GBM tissues and five samples from adjacent normal tissues. Two independent microarray datasets from the GEO (Gene Expression Omnibus, RRID:SCR_005012) of the National Center for Biotechnology Information (NCBI, RRID:SCR_006472) were used to further narrow down these DEGs. GSE68848 (Madhavan et al., 2009) included the gene expression data of 228 GBM tissue samples and 28 samples from adjacent normal tissues, whereas GSE4290 (Sun et al., 2006) included 77 GBM tissue samples and 23 adjacent normal tissue samples (Table 1). All these samples from GEO were included on the Affymetrix Human Genome U133 Plus 2.0 Array platform. Since nontransformed gene expression values usually are substantially skewed in linear scale, we performed data normalization of the two datasets to obtain equal distributions of the probe signal intensities suitable for the analysis using the R package “limma” (LIMMA, RRID:SCR_010943). Expression values for all genes were transformed to the log base 2. DEGs were selected via the same standard as the TCGA dataset. The final set of DEGs was obtained via overlapping the three datasets above.

**Table 1:** The characteristics of the datasets used for screening differentially expressed genes

Dataset	Sample		Tissue	Method
	GBM	Control		
TCGA	168	5	GBM	Illumina RNA Sequencing
GSE68848	228	28	GBM	Affymetrix Human Genome U133 Plus 2.0 Array
GSE4290	77	23	GBM	Affymetrix Human Genome U133 Plus 2.0 Array

### 2.3 Enrichment analysis of GO function and Kyoto Encyclopedia of Genes and Genomes pathway

Within the selected DEGs, we performed the functional enrichment analysis of Gene Ontology (GO) function and Kyoto Encyclopedia of Genes and Genomes (KEGG) pathways using the WebGestalt (WebGestalt: WEB‐based GEne SeT AnaLysis Toolkit, RRID:SCR_006786) via a significance threshold of FDR less than 0.05 to understand the critical biological implications of the identified DEGs in GBM tissues.

### 2.4 Selection of best genes for modeling

After obtaining the DEGs associated with OS, we used a robust likelihood‐based survival approach to select the best genes for modeling. The analysis was performed in R environment using the R package “rbsurv” (rbsurv, RRID:SCR_001175). Our detailed algorithm is summarized as follows:

1. All the TCGA GBM samples were randomly divided into the training set with N*(1 − *p*) samples and the validation set with N*p samples (*p* = 1/3). Next, a gene was fitted to the training set of samples using the Cox proportional hazards model and the parameter estimate for this gene was obtained. Log likelihood was evaluated with the parameter estimate and the validation set of samples. This evaluation was performed for each gene.
2. The above procedure was repeated 10 times, thus 10 log likelihoods were obtained for each gene. Next, the best gene g_(1)_ with the largest mean log likelihood was selected. All the best survival‐associated genes were selected by the robust likelihood‐based approach.
3. Let g_(1)_ be the selected best gene in the previous step. Adjusting for g_(1)_, the next best gene was found by repeating the above two steps. In other words, g_(1)_ + g_(j)_ was evaluated for every j and an optimal two‐gene model, g_(1)_ + g_(2)_, was selected. This forward gene selection procedure was continued until fitting was impossible because of the lack of samples. Thus, a series of K models were generated: M_1_ = g_(1)_, M_2_ = g_(1)_ + g_(2)_, …, M_K−1_ = g_(1)_ + g_(2)_ + … + g_(K − 1)_, M_K_ = g_(1)_ + g_(2)_ + … + g_(K)_.
4. To avoid over‐fitting, Akaike information criterion (AICs) for all the candidate models were computed, and an optimal model with the smallest AIC was selected. The model that is best according to AIC is the one that minimizes prediction error (Akaike, 1981; Cho, Yu, Kim, & Kang, 2008).

### 2.5 Development of the gene prognostic nomogram

We used the R package “rms” to generate the prognostic nomogram based on the expression level of the genes selected by the previous step, using the 168 training samples from TCGA. In the package, the “cph” function was used to build the COX model. Based on the model, the “nomogram” function was used to generate the prognostic nomogram. The length of line segments corresponding to each variable in the prognostic nomogram reflects the contribution of predictors to the patient outcome.

### 2.6 External validation of the gene prognostic nomogram

After the nomogram was generated, an independent dataset GSE43378 (Kawaguchi et al., 2013) (Affymetrix Human Genome U133 Plus 2.0 Array; *N* = 32) with complete OS information was used as the external validation dataset. For this cohort, we calculated the C‐index to test the ability of the gene prognostic nomogram to discriminate the outcome of patients. To evaluate the calibration of the gene prognostic nomogram, we also generated calibration curves for the 12‐, 15‐ and 18‐month survival predictions, as well as survival curves for the high‐risk group and low‐risk group. To discover whether our gene nomogram could be deemed as an independent prognostic factor, multivariate Cox regression was performed with the nomogram, patient age, and gender.

## 3 RESULTS

### 3.1 Genes associated with OS

The TCGA dataset included 168 effective samples with expression values for 29,846 genes. Additionally, all datasets contain information on sample observation time and censoring status. We preliminarily identified 2,154 survival associated genes with a *p* < 0.05, using log‐rank test.

### 3.2 DEGs in GBM and adjacent normal tissues

**Figure 1:**
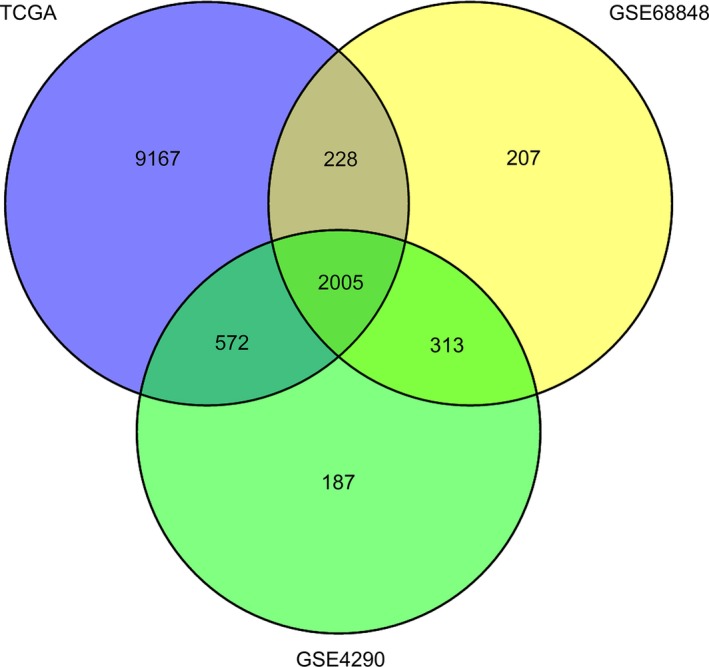
The 2,005 differentially expressed genes (DEGs) were identified by overlapping the TCGA cohort, GSE68848 cohort and GSE4290 cohort using Venny 2.1.0. The criteria of DEGs was set as the thresholds of fold change greater than 1 or less than −1 and adjusted *p* < 0.05

**Figure 2:**
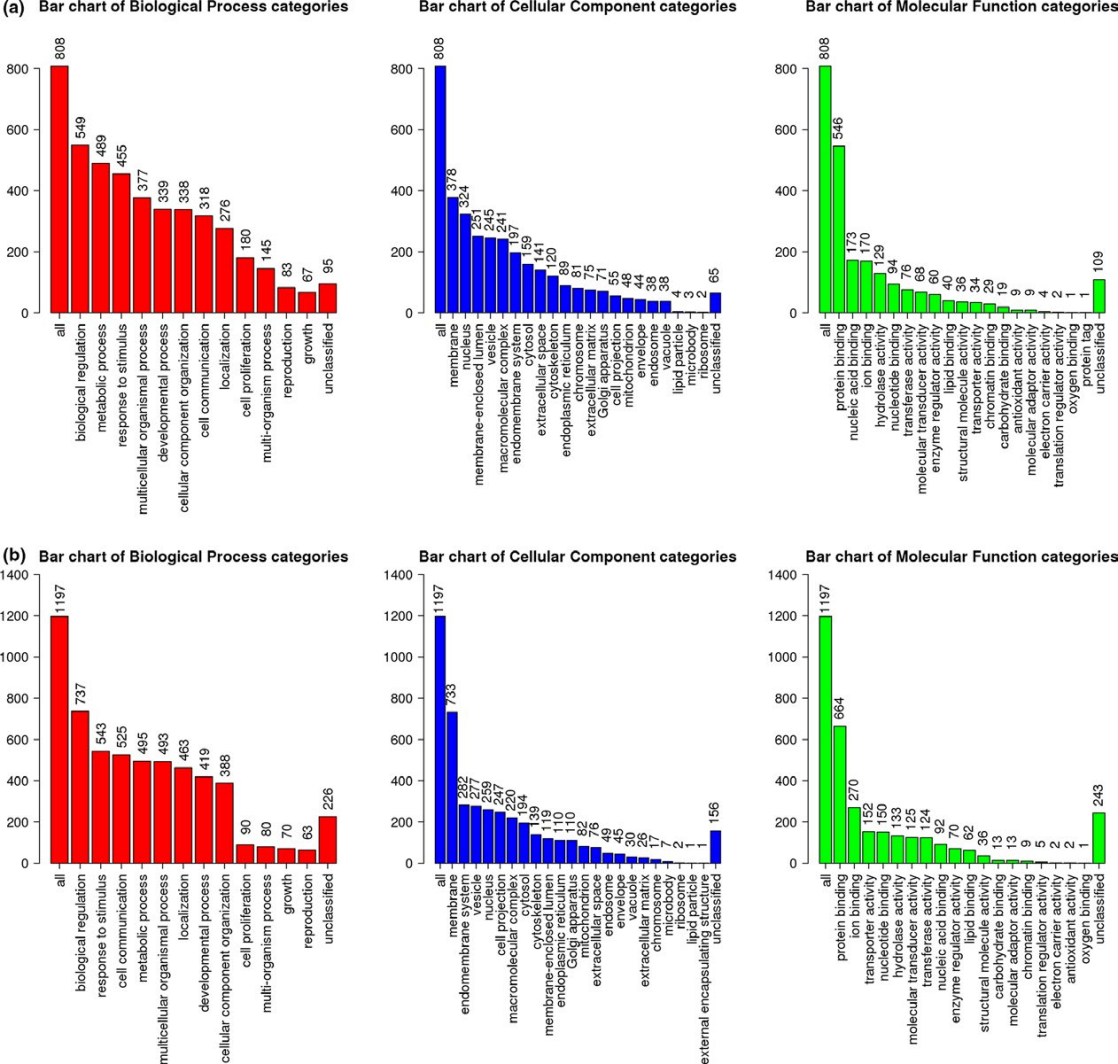
GOSlim summary for the differentially expressed genes (DEGs). Each Biological Process, Cellular Component and Molecular Function category is represented by a red, blue and green bar, respectively. The height of the bar represents the number of DEGs observed in the category. Figure 2a shows the GO terms of upregulated genes. Figure 2b shows the GO terms of downregulated genes

We compared the expression values of these 2,154 genes among 168 samples from GBM tissues and five samples from adjacent normal tissues in TCGA datasets. DEGs(11,972) were initially identified with the thresholds of fold change greater than 1 or less than −1 and adjusted *p* < 0.05. To further narrow down to a list of more effective DEGs, we screened DEGs in two independent datasets (GSE68848 included 228 samples from GBM tissues and 28 from adjacent normal tissues; GSE4290 included 77 samples from GBM tissues and 23 from adjacent normal tissues) using the same criteria. In the GSE68848 cohort, 2,753 DEGs were identified. In the GSE4290 cohort, 3,077 DEGs were identified. After overlapping these three datasets, 2,005 DEGs were identified to exhibit different expression between GBM tissues and adjacent normal tissues, including 808 upregulated genes and 1,197 downregulated genes (Figure 1). To understand the biological implications of the identified DEGs in GBM tissues, we performed enrichment analysis of GO function and KEGG pathways within the DEGs. The GO terms of upregulated genes and downregulated genes are presented in Figure 2, respectively. The top 10 enriched KEGG pathway terms of upregulated genes and downregulated genes are provided in Tables 2 and 3, respectively.

**Table 2:** The top 10 enriched KEGG pathway terms of upregulated DEGs

KEGG ID	KEGG pathway	No. of genes	FDR
hsa04110	Cell cycle	28	1.45E−09
hsa04610	Complement and coagulation cascades	21	1.39E−08
hsa05150	Staphylococcus aureus infection	16	5.38E−07
hsa05166	HTLV‐I infection	36	5.48E−07
hsa04512	ECM‐receptor interaction	19	5.48E−07
hsa04115	p53 signaling pathway	16	6.88E−06
hsa04145	Phagosome	24	1.57E−05
hsa05133	Pertussis	16	2.13E−05
hsa05205	Proteoglycans in cancer	28	2.14E−05
hsa05416	Viral myocarditis	12	6.57E−04

Note

DEGs, differentially expressed genes; KEGG, Kyoto Encyclopedia of Genes and Genomes.

**Table 3:** The top 10 enriched KEGG pathway terms of downregulated DEGs

KEGG ID	KEGG pathway	No. of genes	FDR
hsa04723	Retrograde endocannabinoid signaling	34	0
hsa04727	GABAergic synapse	30	0
hsa05032	Morphine addiction	29	1.46E−13
hsa05033	Nicotine addiction	19	1.92E−12
hsa04724	Glutamatergic synapse	30	8.75E−12
hsa04921	Oxytocin signaling pathway	34	1.10E−10
hsa05031	Amphetamine addiction	22	1.30E−10
hsa04721	Synaptic vesicle cycle	21	1.77E−10
hsa04020	Calcium signaling pathway	36	1.77E−10
hsa04728	Dopaminergic synapse	30	1.77E−10

Note

KEGG, Kyoto Encyclopedia of Genes and Genomes; DEGs, differentially expressed genes.

### 3.3 Best genes for modeling

Of the overlapping 2,154 genes associated with OS and 2,005 DEGs, 168 genes were identified for the final analysis. Using a partial likelihood of the Cox proportional hazard regression model, we next selected the best survival‐associated genes. We implemented a cross‐validation technique considering the large data variability. A forward selection was employed to generate a series of gene models, and the optimal model was then selected using the minimal AIC. Finally, five genes (OSMR, BICDL1, SH3BP2, MSTN, and RGS14) were selected that can optimally predict the OS of GBM patients (Table 4).

**Table 4:** The best genes predicting overall survival of glioblastoma patients

Gene symbol	nloglik	AIC	Selected
OSMR	466.84	935.69	*
BICDL1	459.69	923.38	*
SH3BP2	455.95	917.90	*
MSTN	453.84	915.68	*
RGS14	452.75	915.50	*

*Gene selected for the optimal model.

### 3.4 The development of a prognostic nomogram

**Figure 3:**
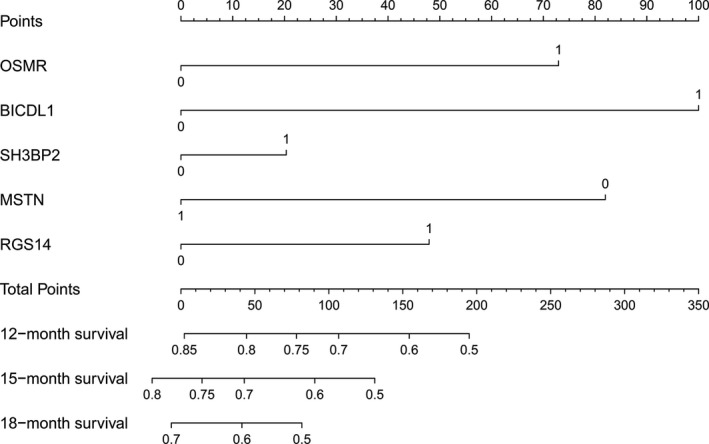
The 5‐gene prognostic nomogram based on the expression level of OSMR, BICDL1, SH3BP2, MSTN, and RGS14. The high and low expression levels of each gene were represented by “1” and “0” respectively. “Points” is the score corresponding to the expression level of a single gene. “Total points” is the sum of the “Points” which five genes get. The higher “Total points” value means the lower survival probability of GBM patients

We used the R package “rms” to generate the prognostic nomogram based on the expression level of the five genes (OSMR, BICDL1, SH3BP2, MSTN, and RGS14). As shown in Figure 3, "1" represents the high expression level of each gene, whereas "0" represents the low expression level of each gene. “Points” is the score corresponding to the expression level of a single gene. “Total points” is the sum of the “Points” that five genes get, which corresponds to the accurate survival probability of each sample. An increased “Total points” indicates greater mortality risk for GBM patients.

### 3.5 External validation of the prognostic nomogram

**Figure 4:**
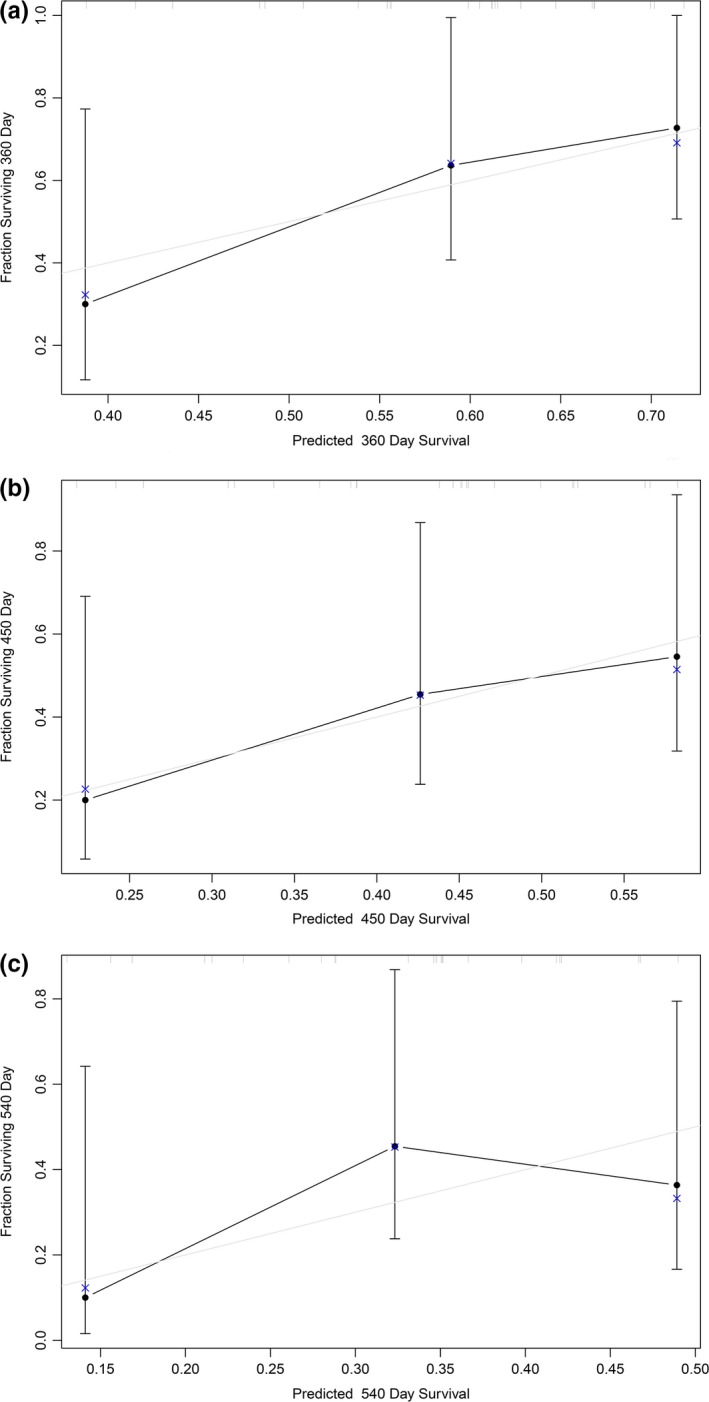
Performance of 5‐gene prognostic nomogram in predicting survival probability of GBM patients from GSE43378 cohort. (a) The calibration curve was generated for 12‐month survival predictions of the 5‐gene prognostic nomogram. (b) The calibration curve was generated for 15‐month survival predictions of the 5‐gene prognostic nomogram. (c) The calibration curve was generated for 18‐month survival predictions of the 5‐gene prognostic nomogram

**Figure 5:**
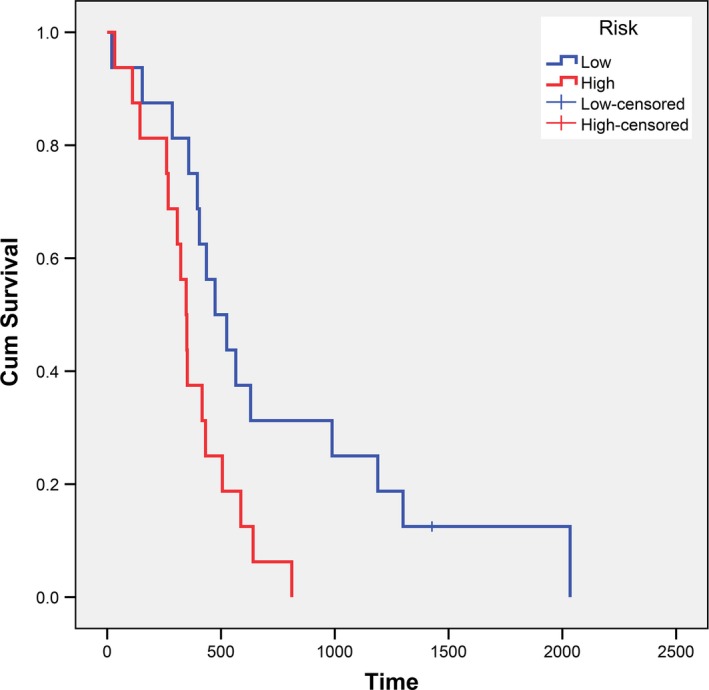
The survival curves of the high risk group and low risk group of GSE43378 cohort divided by 5‐gene prognostic nomogram. The high risk group exhibited a poorer prognosis compared with the low risk group (*p* = 0.018)

**Figure 6:**
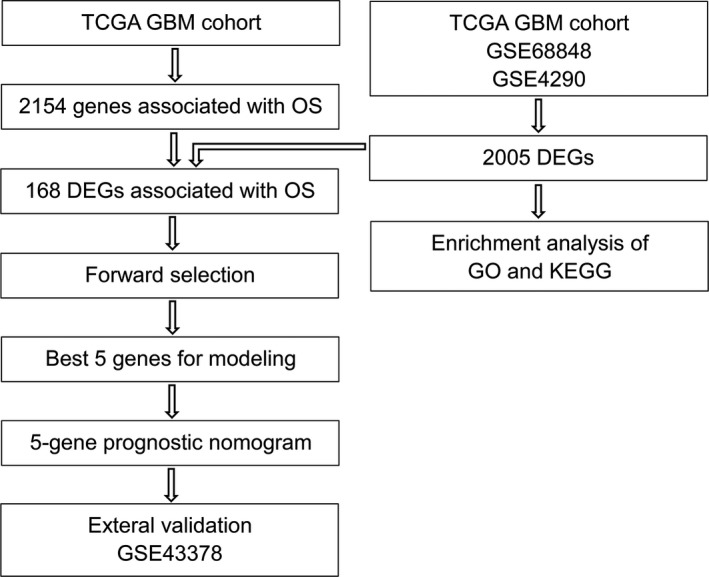
The process of developing the 5‐gene prognostic nomogram. First, 168 differentially expressed genes associated with the OS in GBM patients were identified by univariate survival analysis and differential expression analysis. Next, a robust likelihood‐based survival modeling approach was applied to identify the best genes for prognosis prediction. Then, the gene prognostic nomogram was generated based on five genes (OSMR, BICDL1, SH3BP2, MSTN, and RGS14). Finally, the 5‐gene prognostic nomogram was validated in an independent cohort on different platforms

An independent cohort of GBM patients with OS and gene expression from different platform was used to evaluate the robustness and effectiveness of the gene prognostic nomogram. The validation data set GSE43378 was obtained using Affymetrix Human Genome U133 Plus 2.0 Array and 32 GBM samples with complete data regarding observation time and censoring status. We next calculated the C‐index (C‐index = 0.629, *p* = 0.0273) and generated the calibration curve for the 12‐, 15‐ and 18‐month survival predictions to evaluate the calibration of the gene prognostic nomogram shown in Figure 4. These results demonstrate the ability of our gene prognostic nomogram to discriminate the outcome of patients and the calibration between the probabilities of the actual outcome and prediction. On the other hand, the cohort was divided into a high risk group and a low risk group using the nomogram. Noteworthily, the survival curve revealed that the high risk group exhibited a poorer prognosis compared to the low risk group (*p* = 0.018, Figure 5). Multivariate Cox regression was performed with the gene nomogram, patient age and gender in the validation dataset. As a result, only the gene nomogram was in the equation (*p* = 0.021, Exp(B) = 2.506), which showed that it could work as a prognostic factor independent of age or gender. Therefore, the prognostic nomogram based on five genes could effectively predict the survival probability of patients with GBM. The process of this study is represented in Figure 6.

## 4 DISCUSSION

In this study, we developed a 5‐gene prognostic nomogram that exhibits the ability of predicting the survival probability for patients within GBM. Using this tool, we could predict patients with higher risk of mortality, and thus a need for them to get more immediate attention and treatment. Within the survival analysis of the GBM samples from TCGA, a total of 2,154 genes were identified to be associated with the OS of GBM patients. Meanwhile, 2,005 genes were found differentially expressed between GBM tissues and adjacent normal tissues by analyzing the TCGA dataset and two other independent datasets. By KEGG analysis, we found that these DEGs were enriched in the signaling pathways such as cell cycle, p53 signaling pathway, retrograde endocannabinoid signaling and dopaminergic synapse. A previous study showed that the cell cycle and p53 signaling pathways co‐mutated in GBM (Wei, Wang, & Zhao, 2014). Notably, the p53 signaling pathway exerts an important role in glioma pathogenesis (Stegh & DePinho, 2011). After identifying the overlapping the genes associated with OS and DEGs, we further selected the best five genes to develop the gene prognostic nomogram: OSMR, BICDL1, SH3BP2, MSTN, and RGS14.

Recently, a study demonstrated that OSMR is required for GBM tumor growth. The study discovered that the cytokine receptor OSMR is an essential co‐receptor of EGFRvIII that plays a prominent role in GBM tumorigenesis, and it is also a highly upregulated direct transcriptional target gene of STAT3 in GBM (Jahaniasl, Yin, & Soleimani, 2016). Similarly, another study identified OSMR as a novel key regulator of brain tumor stem cell proliferation and GBM tumorigenesis (Mohan, Bonni, & Jahani‐Asl, 2017). Their findings highlight the significance of OSMR as a potential druggable target in GBM therapy. Furthermore, our study not only validated the relationship between OSMR and GBM but also demonstrated the effectiveness of using OSMR on the prognosis of GBM.

SH3BP2, a c‐Abl binding protein in mice and humans (Bell, Shaw, Jou, Myers, & Knowles, 1997; Ren, Mayer, Cicchetti, & Baltimore, 1993), is expressed in most cell types (Reichenberger, Levine, Olsen, Papadaki, & Lietman, 2012). SH3BP2 acts as an adapter protein to control intracellular signaling by interacting and forming complexes with binding proteins (Deckert & Rottapel, 2006; Le et al., 2004) and scaffolding proteins (Le, Moon, Foucault, Breittmayer, & Deckert, 2007). MSTN, a potent negative regulator of muscle growth, is indispensable in regulating neuronal and muscle function (Augustin et al., 2017). Inhibition of MSTN is a promising method to alleviate muscle wasting, especially considering that loss of functional myostatin in humans is not related with apparent deleterious effects other than muscle hypertrophy (Schuelke et al., 2004). Currently in clinical trials, applying monoclonal antibodies that bind to myostatin to inhibit its function is a potential therapy to treat cachexia syndrome in cancer patients (Nicole, Lisa, Wolfram, Anker, & Stephan, 2014). RGS14, a multifunctional scaffolding protein, integrates heterotrimeric G protein and H‐Ras signaling pathways (Vellano, Brown, Blumer, & Hepler, 2012), and plays a key role in regulating synaptic plasticity (Branch & Hepler, 2017). However, the function of BICDL1 remains unknown.

Except OSMR, the other four genes have not been reported in previous GBM studies, indicating that these genes could represent potential target genes for GBM treatments, and their biological roles in the development of GBM would be of great interest in further studies.

The 5‐gene prognostic nomogram was validated using an independent dataset on different platform. The C‐index revealed the nomogram's ability to discriminate the outcome of patients; The calibration curve presented the calibration between the probability of the actual outcome and the probability of prediction; The result of multivariate Cox regression showed that the nomogram could work as an independent prognostic factor. All these above findings demonstrated that this prognostic nomogram based on five genes has the capability of predicting the survival probability of GBM patients. On the other hand, we have considered trying to build a new model including patients’ age. However, after adjustment for age, we obtained another 9‐gene model whose prediction power is not as good as the 5‐gene model. Finally, the 5‐gene model was chosen.

In prior cancer studies, the risk score system was often constructed to predict the mortality risk for patients. However, in clinical practice, risk scores occasionally do not accurately reflect the survival probability. It is important to build a convenient predicting tool for clinical practice. Our study developed a prognostic nomogram with five genes that could effectively predict the survival probability for GBM patients. Moreover, the method is flexible and convenient. The result is presented by relative risk combined with absolute survival probability, which is more visual and intuitive. Based on the relative risk ratio, the specific survival probabilities of the individuals can be queried according to the level of the five genes, and patients predicted to be in severe condition will very likely need more attention and care. The prognostic nomogram can be easily generated using R software and can serve as a powerful tool in model prediction. It is important to make Real‐time Quantitative PCR (QPCR) assay more popular in clinical practice. The expression level of genes could be obtained with QPCR, which could make our gene nomogram implemented into routine clinical setting conveniently. This model will provide a good reference for cancer researchers. In our future studies, we will collect more clinical tumor tissues which are used to determine the cut‐off point of risk. Meanwhile, other well‐known clinical prognostic factors that could not be obtained from the database, like IDH mutation and pre‐operative KPS, should be the key point in our further study. With the patient's clinical information collected more comprehensively, we will try to build a model with adjustment for these well‐known risk factors or find a way to combine our nomogram with these clinical characteristics.

## CONFLICT OF INTEREST

The authors declare no competing interests.
